# Double-Barrel Urocolostomy After Pelvic Exenteration: Short-Term Morbidity and Patient-Reported Quality of Life

**DOI:** 10.1245/s10434-025-17020-6

**Published:** 2025-03-14

**Authors:** L. J. van Kesteren, L. R. Moolenaar, J. A. Nieuwenhuijzen, V. de Bruijn, O. C. Moldovan, M. S. Vlug, W. Lameris, R. Hompes, J. B. Tuynman

**Affiliations:** 1 Department of Surgery, Amsterdam UMC, Vrije Universiteit Amsterdam, Amsterdam, The Netherlands; 2 Treatment and Quality of Life and Imaging and Biomarkers, Cancer Center Amsterdam, Amsterdam, The Netherlands; 3 Department of Urology, Amsterdam UMC, Vrije Universiteit Amsterdam, Amsterdam, The Netherlands

**Keywords:** Double barrel urocolostomy, Double barrel (wet) colostomy, DBUC, Total pelvic exenteration, Recurrent rectal cancer

## Abstract

**Background:**

Total pelvic exenteration is a radical surgical procedure for advanced pelvic malignancies. Traditionally, an ileal conduit is created on the right abdominal wall for urinary diversion and an end-colostomy on the left abdominal wall for fecal diversion. However, this approach is associated with increased morbidity and a negative impact on quality of life (QoL). A unilateral double-barrel urocolostomy (DBUC) offers an alternative using the sigmoid colon for urinary drainage. This can potentially reduce complications, improve QoL, and preserve the right vertical rectus abdominis muscle (VRAM) flap for pelvic reconstruction. This study aimed to evaluate the impact of the DBUC on 90-day morbidity and QoL of patients undergoing pelvic exenteration for locally advanced colorectal and anal cancer.

**Methods:**

Data were prospectively collected from all patients who underwent pelvic exenteration with DBUC reconstruction for colorectal and anal cancer at our tertiary care center between January 2020 and May 2023.

**Results:**

This study enrolled 20 patients. Postoperative complications were observed in 19 patients, including seven major complications. Two complications were directly attributable to the DBUC. Patients reported favorable QoL outcomes in terms of global health, functional ability, and symptom management, with expected limitations in physical performance due to extensive abdominal surgery. At 1 year after surgery, all the patients preferred the DBUC over separate bilateral ostomies.

**Conclusion:**

The DBUC procedure has demonstrated safety and efficacy in terms of short-term morbidity and favorable patient-reported QoL, making it an attractive alternative to dual ostomies for patients undergoing pelvic exenteration, particularly when VRAM reconstruction is considered.

**Supplementary Information:**

The online version contains supplementary material available at 10.1245/s10434-025-17020-6.

Total pelvic exenteration is a radical surgical procedure reserved for locally advanced primary or recurrent pelvic malignancies without distant metastases. This procedure involves en bloc resection of the pelvic organs, including the reproductive organs, bladder, and rectosigmoid, requiring both urinary and fecal diversion. Although various techniques have been described,^1^ the most common urinary diversion is a uretero-ileal anastomosis using a right-sided ileal conduit, as described by Bricker.^2^

To address morbidity and impact on quality of life (QoL) associated with a second stoma, Brunschwig^3^ introduced the double-barrel wet colostomy (DBWC) in 1948. This technique involves a bilateral ureterosigmoidostomy connected to an end-colostomy, mixing urine and feces before they exit the opening. Despite its initial promise, the DBWC has been associated with significant complications, including pyelonephritis and carcinogenesis at the ureterosigmoidostomy site.^4^

In 1989, Carter et al.^5^ introduced the double-barrel urocolostomy (DBUC) as an alternative approach for simultaneous urinary and fecal diversion. This technique uses the sigmoid colon as a urinary reservoir, eliminating the need for an additional ileal anastomosis and creating a single-site double ostomy. As mixing of urine and feces occurs externally within the ostomy bag, the DBUC may reduce the risk of urinary tract infections and colonic dysplasia.

Pelvic exenteration is often combined with reconstructive surgery to fill the pelvic dead space with healthy viable tissue, thereby reducing the risk of postoperative complications. In recent years, flap reconstruction techniques have gained popularity because of their ability to minimize infections, wound dehiscence, and fistula formation. A vertical rectus abdominis muscle (VRAM) flap is the gold standard for pelvic reconstruction in patients with extensive pelvic floor defects. A key advantage of the DBUC is that it preserves the ability to harvest the right VRAM flap, unlike the traditional two-ostomy approach. In addition, from a patient perspective, a single left-sided stoma is often preferred over the management of two separate ostomies because it simplifies stoma care and improves the overall QoL.

This cohort study aimed to evaluate the impact of the DBUC on short-term morbidity and patient-reported QoL of patients undergoing pelvic exenteration for locally advanced primary or recurrent colorectal and anal cancer at our tertiary care center.

## Methods

### Patient Selection

Data from all patients who underwent pelvic exenteration with DBUC reconstruction for colorectal and anal cancer between January 2020 and May 2023 at our tertiary care center were retrospectively analyzed after extraction from a prospective, anonymized database. Eligible patients were informed of the available reconstructive options, including the standard technique (right-sided ileal conduit for urinary diversion and left-sided end-colostomy) and the DBUC technique. They also consulted with a specialized ostomy nurse to discuss the daily implications of each technique.

### Outcome Measures

The primary outcome was 90-day postoperative complications related to the DBUC, classified according to the Clavien-Dindo (CD) scale. Minor morbidity was defined as CD grade ≤ 2 and major morbidity as CD grade ≥ 3. Urinary tract infection (UTI) was defined as the presence of clinical symptoms indicative of pyelonephritis, accompanied by elevated inflammatory markers and requiring antibiotic therapy. Sepsis was defined as either the presence of positive blood cultures or a diagnosis documented by the surgical team based on clinical suspicion, necessitating intravenous antimicrobial therapy. Urinary leakage was defined as leakage from the ureter-sigmoid anastomosis, confirmed clinically or radiologically, requiring percutaneous or surgical intervention. Anastomotic leakage was defined as leakage from the entero-enteral anastomosis, confirmed clinically or radiologically, necessitating percutaneous or surgical management. Ureteroenteric stricture was defined as radiographic obstruction at the site of the ureter-sigmoid anastomosis and clinical symptoms including pain, infection, and deteriorating renal function. Secondary outcomes included reinterventions, readmissions, ureteral conduit leakage, and QoL. Quality of life was assessed using the European Organization for Research and Treatment for Cancer Quality of Life Questionnaire (EORTC QLQ-C30, EORTC QLQ-CR29), the Short-Form Health Survey 36 version 2 (SF-36v2), the EuroQol - five dimensions - five levels questionnaire (EQ-5D-5L), the Dutch Stoma-QoL questionnaire, and the Decision Regret Scale.^6–10^

### Surgical Procedure

**Fig. 1:**
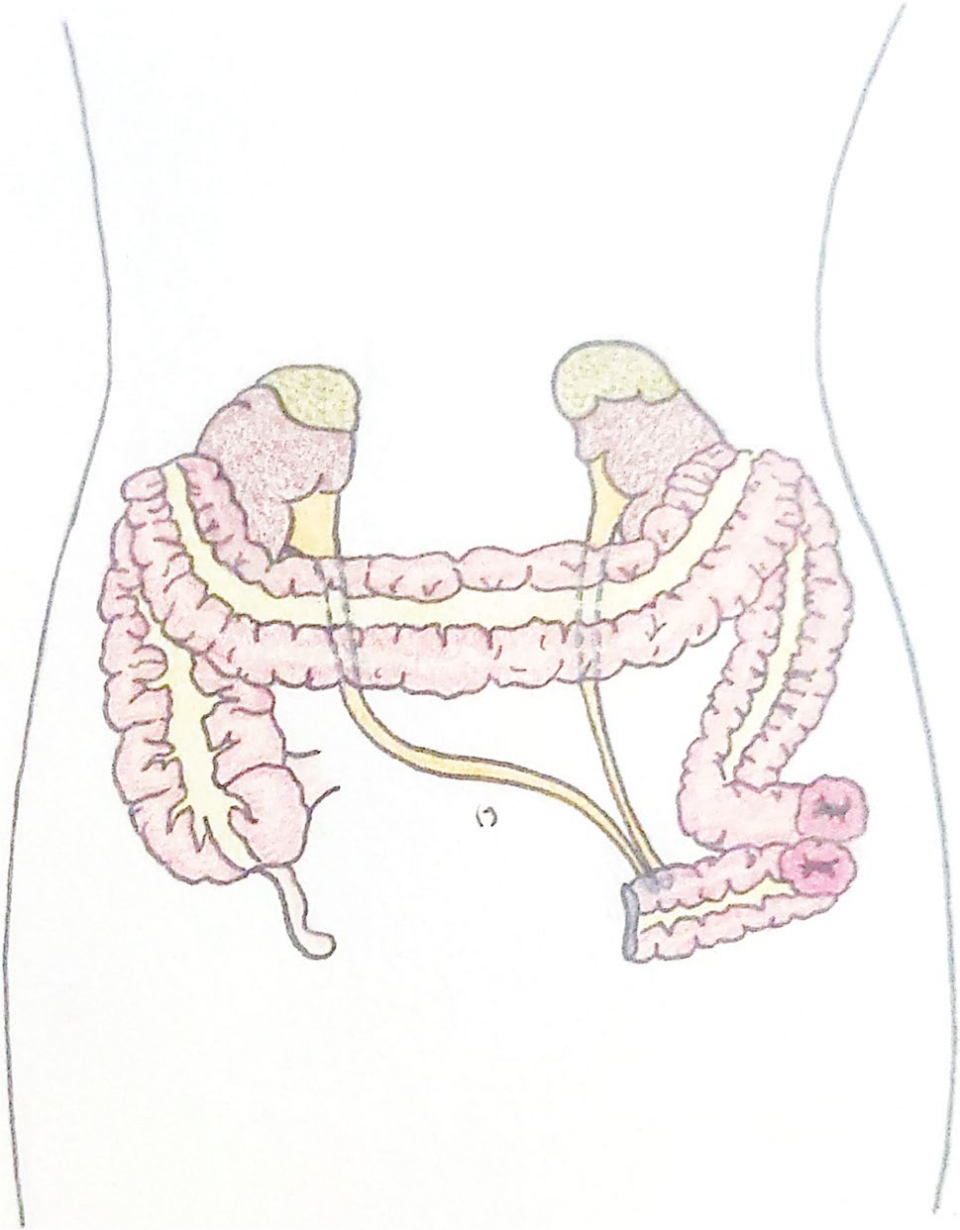
Double-barrel urocolostomy (DBUC)

All the patients underwent complete bowel preparation before surgery. Procedures were performed using either a minimally invasive robotic approach or an open approach. The left hemicolon was mobilized, including high ligation of the mesenteric vein if necessary, to achieve a sufficient length for the construction of a tension-free urinary conduit and colostomy. The distal descending colon was carefully transected to preserve the integrity of the mesocolon and ensure optimal vascularization of the urinary conduit. The colon was transected, leaving 10–15 cm of the sigmoid distal to the planned stoma site to serve as the urinary reservoir and conduit for the DBUC. The ureters were mobilized, ligated, and transected to provide sufficient length for the anastomosis. The right ureter was redirected retroperitoneally to the left side. The DBUC was constructed by anastomosing the ureters side-to-side over single-J stents to the urinary conduit and passed through the abdominal wall to create a stoma (Fig. 1). If necessary, the right VRAM flap was harvested through an open approach for pelvic reconstruction.

### Statistics

Categorical data are reported as frequencies and percentages. Numeric data are presented as either the mean with standard deviation (SD) or median with interquartile range (IQR), depending on the distribution. All analyses were performed using IBM SPSS Statistics version 28 (IBM, Chicago, IL, USA). A two-tailed *p* value lower than 0.05 was considered statistically significant.

## Results

### Patient and Surgical Characteristics

Between 31 August 2018 and 3 April 2023, 20 patients underwent total pelvic exenteration with DBUC reconstruction at our tertiary care center. The median age was 63.2 years (IQR, 55.3–75.7 years). All the patients were in good physical condition (WHO performance status 0 or 1). Of the 20 patients, 3 underwent minimally invasive pelvic exenteration, and 17 received an open surgical approach. A left-sided DBUC was performed for 18 patients, whereas the remaining 2 patients received a left-sided ileal conduit with an end-colostomy constructed as a single-site double-barrel ostomy. Three patients required resection of an additional bowel segment as part of the oncologic resection, resulting in an additional anastomosis. Approximately 45% of the patients underwent pelvic reconstruction with a VRAM flap. For smaller defects, reconstruction included gluteal transposition flaps, lotus paddle flaps, and primary closure with or without mesh reinforcement. No intraoperative complications other than blood loss were reported (Table 1).

**Table 1:** Patient and surgical characteristics

	(*n* = 20) *n* (%)
Median age: years (IQR)	63.2 (55.3–75.7)
Sex	
Male	16 (80.0)
Female	4 (20.0)
Median BMI: kg/m^2^ (IQR)	25.2 (22.1–29.3)
ASA	
2	15 (75.0)
3	5 (25.0)
Primary tumor	
Rectum	16 (80.0)
Sigmoid	3 (15.0)
Anus	1 (5.0)
Previous abdominal surgery	
Yes	16 (80.0)
No	4 (20.0)
Ostomy *in situ*	
Colostomy	10 (50.0)
Bricker ileal conduit and colostomy	1 (5.0)
Ileostomy	1 (5.0)
None	10 (40.0)
Type of surgery	
Laparoscopy	1 (5.0)
Laparotomy	19 (95.0)
Type of urostomy	
Urosigmoidocutaneostomy	18 (90.0)
Uroileocutaneostomy	2 (10.0)
Revision previous ostomy	
Yes	4 (20.0)
No	16 (80.0)
Bowel anastomosis	
Yes, colon	3 (15.0)
Yes, ileum	2 (10.0)
No	12 (75.0)
VRAM	
Yes	9 (45.0)
No	11 (55.0)
Median operating time: min (IQR)	540 (450–600)
Median blood loss: ml (IQR)	1000 (350–1424)

IQR, interquartile range; BMI, body mass index; ASA, American Society of Anesthesiologists; VRAM, vertical rectus abdominis myocutaneous

### Postoperative Outcomes

The postoperative outcomes are described in Table 2. The median hospital stay was 13 days (IQR 10-24). Postoperative complications occurred for 19 patients (95%). Seven patients (35%) experienced major complications. Two complications were related to the urinary diversion, including one ureteral leak and one urinoma requiring radiologic drainage. Other major complications included two cases of intra-abdominal sepsis, one case of systemic inflammatory response syndrome (SIRS), one case of colonic anastomotic leak, and one case of COVID-19-related death. Three patients experienced UTIs requiring antibiotic treatment within 90 days after surgery. Ten patients (50%) were readmitted, including three who were readmitted multiple times: one patient for recurrent UTI, a second patient for anastomotic leakage after resection of an additional bowel segment as part of the oncologic resection, and a third patient for diagnostic purposes due to persistent urinary leakage at the ureterosigmoidostomy site and recurrent UTI at 90 days. The last patient also reported a prolonged primary hospital stay due to repeated VRAM flap failure. No ureteroenteric anastomotic strictures were reported. The 30-day postoperative mortality rate was 0%, although one patient died at 47 days.

**Table 2:** Postoperative outcomes <90 days

	(*n* = 20) *n* (%)
Median follow-up: months (IQR)	7.9 (4.2–21.7)
Median hospital stay: days (IQR)	13 (10–24)
Post-operative ICU admission	
Yes	2 (10.0)
No	18 (90.0)
Postoperative complications	
CD≥3a	7 (35.0)
CD<2	12 (60.0)
No complications	1 (5.0)
DBUC-related complications	
CD≥3a	2 (10.0)
CD<2	8 (40.0)
None	10 (50.0)
Ureter-conduit leakage	
Yes	2 (10.0)
No	18 (90.0)
Ureteroenteric stricture	
Yes	0 (0.0)
No	20 (100.0)
Recurrent urinary infections	
Yes	3 (15.0)
No	17 (85.0)
Reintervention <90 days	
Yes	2 (10.0)
No	18 (90.0)
Revision of DBUC	
Yes	3 (15.0)
No	17 (85.0)
No. of readmissions	
0	10 (50.0)
1	7 (35.0)
2	2 (10.0)
3	1 (5.0)
In-hospital mortality <30 days^a^	
Yes	0 (0.0)
No	20 (100.0)

IQR, interquartile range; ICU, intensive care unit; Clavien-Dindo scale; DBUC, double-barrel urocolostomy

^a^One patient was admitted to the ICU and died after 47 days due to a COVID-19 infection.

### Quality of Life

Quality-of-life questionnaires were completed by 14 patients at a median follow-up time of 9 months (IQR, range 4.3-23.2 months) after surgery. Not all the patients had QoL data available, including three patients who died: one due to a COVID-19 infection and two due to recurrent disease (respectively 16.7 and 24.2 months after surgery).

### EORTC QLQ-C30

Overall, the patients reported a favorable QoL in terms of global health, functional ability, and symptom management (Table 3). The majority of the patients (77.6%) experienced difficulties with role functioning (66.7 ± 25.1, Supplementary Table 1), which included the ability to manage work, hobbies, and/or leisure activities. The most commonly reported problems were fatigue, insomnia, and pain.

**Table 3:** EORTC QLQ-C30 results

Scaling (*n* = 14)	Item no.	Mean ± SD	Range
Global health status/QoL (higher score indicates better status)	29, 30	77.4 ± 16.2	50–100
Functional scales (higher score indicates better function)	1–7, 20–27	81.7 ± 10.6	66.7–100
Symptom scales/items (higher score indicates more problems)	10–19, 28	13.2 ± 12.2	0–38.5

EORTC, European Organisation for Research and Treatment of Cancer; QLQ, quality-of-life questionnaire; SD, standard deviation; QoL, quality of life

### EORTC QLQ-CR29

In terms of urinary and fecal diversion, QoL was primarily affected by high urinary frequency, which could be interpreted as rapid filling of the ostomy bag. Colostomy-related problems, such as unwarranted gas leakage, fecal leakage, and care-related problems, were less prominent (Supplementary Table 2). The EORTC QLQ-CR29 scores are presented in Table 4.

**Table 4:** EORTC CLC-CR29 results^a^

Scaling	*n*	Item no.	Mean ± SD	Range
Urostomy function	7–9	31–34	34.0 ± 27.3	0–100
Colostomy function	9–14	38, 39, 49, 50 52, 53	17.0 ± 19.8	0–50

EORTC, European Organisation for Research and Treatment of Cancer; QLQ, quality-of-life questionnaire; SD, standard deviation

^a^Higher score indicates more problems

### SF36v2

The mean physical and mental health scores of this cohort were 67.0 ± 18.0 and 64.8 ± 13.9, respectively (Table 5). Patients were most likely to experience limitations in performing their work or other strenuous activities due to their physical health (Supplementary Table 3).

**Table 5:** SF36v2 results^a^

	Mean ± SD	Range
Physical component summary (PF + RP + BP + GH)	67.0 ± 18.0	0-100
Mental component summary (VT + SF + RE + MH)	64.8 ± 13.9	0-100

SF36v2, Short-Form Health Survey 36 version 2; SD, standard deviation; PF, physical functioning; RP, role limitations–physical; BP, bodily pain; GH, general health; VT, vitality; SF, social functioning; RE, role limitations–emotional; MH, mental health

^a^Higher score indicates a more favorable health state

### EQ-5D-5L

Within this cohort, the mean EQ-5D-5L index was 0.74 ± 0.19. A significant proportion of patients (85.7%) reported mild to moderate problems with activities of daily living, pain, and mood. One patient experienced extreme pain and discomfort as a complication of partial sacral resection and was referred to the anesthesiology department for personalized pain management. The mean vertical visual analog scale (VAS) score, representing patients’ self-rated health status, was 72 (Supplementary Table 4).

### Stoma-QoL

The mean overall stoma-QoL score obtained by the participants was 56.1 ± 11.3 (0 indicating poor QoL and 100 indicating good QoL). Of the patients surveyed, the majority (93%) would recommend a DBUC to other patients, including one patient who had previously managed two ostomies. One patient did not respond to this question. The most common complaints were an unpleasant smell and concern about rapid filling of the ostomy bag. These issues were reported less frequently by patients who regularly irrigated the fecal conduit and emptied their ostomy bag.

### One-Year Follow-Up Decision Regret Scale

At the 1-year follow-up evaluation, patients were asked if they regretted their decision regarding the DBUC. Of the 16 patients who completed this questionnaire, none expressed regret, and all would recommend the DBUC to other patients. However, qualitative feedback from the patients indicated that the current design of the ostomy bag is not optimal for the larger size and output associated with this type of ostomy.

## Discussion

The current study, representing one of the largest cohorts (*n *= 20) of pelvic exenteration with DBUC reconstruction, demonstrated that a DBUC is a safe and feasible technique with a relatively low incidence of short-term complications, yielding promising QoL outcomes in this challenging patient population. Importantly, at the 1-year follow-up evaluation, none of the patients reported regret regarding their decision to receive a DBUC.

All the patients in this cohort received high doses of pelvic radiation before surgery. Although preoperative radiotherapy is a known risk factor for impaired wound healing and subsequent complications after pelvic exenteration and urinary reconstruction surgery, such as ureteral leakage and stenosis, this study observed overall complication rates similar to those reported in two previous cohort studies.^11–13^ Focusing only on DBUC-specific complications, previous literature documented a twofold higher complication rate.^14^ However, isolating the effect of the DBUC on complication rates is challenging given the significant morbidity associated with pelvic exenteration. Configuration of the DBUC may especially increase the risk of ascending pyelonephritis from the distal urinary reservoir. Although continuous urinary outflow is separated from intermittent stool passage, fecal overflow remains a potential risk.^15^

This study found a higher incidence of UTI in patients with a DBUC than a recent systematic review by Wright et al.^14^ (15% vs 3.7%). However, diagnosing a UTI in DBUC patients is challenging because urinary swabs and cultures are not reliable due to the loss of sterility when collected from the ostomy. In our cohort, all the patients with clinical symptoms suggestive of pyelonephritis and elevated infection markers (i.e., C-reactive protein) requiring antibiotics were reported as having a UTI. In addition, despite patient education emphasizing the importance of regular fecal conduit irrigation and ostomy bag emptying to minimize the risk of overflow, adherence to this regimen was not consistent across patients. These factors may have contributed to the observed discrepancy in UTI incidence. Although not investigated in this study, Nguyen et al.^13^ and Lago et al.^16^ reported no statistically significant differences between conventional ileal conduits and DBUC after total pelvic exenteration.

An advantage of the DBUC is that it avoids the need for an additional ileal anastomosis for urinary diversion. Given the complexity of pelvic exenteration, often involving pre-irradiated patients, avoiding an unnecessary anastomosis may improve patient outcomes. However, our cohort size was insufficient to definitively assess the clinical impact of this approach on recovery and morbidity.

A second advantage of the DBUC is preservation of the integrity of the right abdominal wall. Especially in patients with large perineal defects after pelvic exenteration, robust reconstructive surgery is required to reduce postoperative complications and functional problems related to the pelvic floor.^17^ Unlike traditional ileal conduits, the DBUC facilitates the use of a VRAM flap. In this cohort, a VRAM flap was used for perineal reconstruction in 45% of the patients. Pelvic reconstruction and donor sites can be tailored depending on oncologic resections and residual defects. Less invasive options such as lotus or gluteal flaps may be considered for selected patients. However, a recent systematic review has shown that these techniques may provide a limited volume to adequately cover the pelvic floor and empty pelvis, potentially leading to disadvantages such as reduced mobility, increased risk of wound dehiscence, and higher risk of wound infection.^18^

A third potential advantage of the DBUC is improved QoL and body image due to the presence of a single ostomy compared with a bilateral configuration. This cohort represents the first large series to investigate QoL for patients who underwent pelvic exenteration with DBUC reconstruction. The reported overall QoL in our cohort was 77.4, which is comparable to that observed in patients who received dual ostomies for advanced colorectal and anal cancer (IQR, 67–71.6).^19,20^ Similarly, the EQ-5D index score for our cohort (0.74) was consistent with scores reported for patients who underwent extended abdominoperineal excision for primary or recurrent rectal or anal cancer (0.71). The patients managed with a DBUC scored higher in both physical (67.0 vs 39.90–53.5) and physiologic (64.8 vs 40.1–44.7) domains than the patients with two ostomies.^19,21–25^ Although the patients with a DBUC reported more frequent urostomy-related problems than the patients with double ostomies, particularly faster filling of the ostomy bag, the overall stoma-QoL scores were comparable.^26^ Insomnia was the most common complaint in our cohort, primarily due to an increased frequency of nocturnal voiding, which also has been reported in previous studies.^15,27,28^

A potential complication associated with the DBUC, as reported by one of our patients, is ostomy stacking. This phenomenon is characterized by the accumulation of ostomy appliances due to the lack of ostomy materials specifically designed for the DBUC. This can lead to complications such as skin irritation, leakage, and maceration. Importantly, after a median follow-up period of 9.5 months, none of the patients regretted their decision regarding the DBUC. These findings suggest that a DBUC is a viable option with similar or even superior QoL outcomes compared with dual ostomies in this complex patient population.

In pelvic exenteration, DBUC reconstruction is not widely adopted because of historical concerns regarding the risk of malignancy at the site of ureteral implantation in the sigmoid pouch. However, these concerns are based on older cohorts reporting on the former wet colostomy technique, in which the ureters were implanted in the efferent limb proximal to the colostomy, resulting in direct contact with fecal material at the implantation site. These studies have shown that approximately 40% of patients with a ureterosigmoidostomy are at risk for the development of tumors at the site of their anastomosis, which has probably influenced the reluctance to adopt the DBUC.^29^ However, the proposed double-barrel technique separates urine and faces intraluminally, thereby minimizing the risk of direct contact. In addition, irrigation of the fecal conduit could minimize the risk of fecal-urine mixing, reducing exposure to potentially carcinogenic aromatic amines and nitrosamines. Furthermore, evidence suggests a latency period of 25–30 years for tumor development in these patients, with only 5% progressing to carcinoma.^29,30^ Given the limited life expectancy after pelvic exenteration, as reported by the PelvEx International Collaborative (median overall survival, 37 months), the risk of carcinogenesis in this patient population is negligible.^31^

This study had several limitations, including its non-randomized, non-comparative, single-arm design, small sample size, and short follow-up period. In addition, the QoL outcomes in this cohort may have been significantly influenced by the concomitant effects of pelvic exenteration, potentially masking the true impact of DBUC reconstruction on QoL. The use of tailored questionnaires would provide a more accurate assessment of patient-reported outcomes associated with the DBUC. For future research, we recommend a large prospective cohort study comparing DBUC with ileal conduits, with particular emphasis on long-term outcomes.

## Conclusion

This study showed DBUC to be safe in the context of short-term morbidity, with favorable patient-reported outcomes and high patient satisfaction, offering an attractive alternative to dual ostomies for patients undergoing pelvic exenteration for colorectal and anal cancer, especially when VRAM reconstruction is considered.

## Supplementary Information

Below is the link to the electronic supplementary material.

Supplementary file1 (DOCX 26 KB)

## Data Availability

Research data supporting this publication are available upon request from the corresponding author.

## References

[1] Wood DP Jr. Methods of urinary diversion following radical cystectomy. *J Ky Med Assoc*. 1994;92:96–100. 8035112

[2] Bricker EM. Bladder substitution after pelvic evisceration. 1950. *J Urol*. 2002;167(2 Pt 2):1140–5 (**discussion 6**). 11905889 10.1016/s0022-5347(02)80363-9

[3] Brunschwig A. Complete excision of pelvic viscera for advanced carcinoma; a one-stage abdominoperineal operation with end colostomy and bilateral ureteral implantation into the colon above the colostomy. *Cancer*. 1948;1:177–83. 18875031 10.1002/1097-0142(194807)1:2<177::aid-cncr2820010203>3.0.co;2-a

[4] Brunschwig A, Daniel W. Pelvic exenteration operations: with summary of sixty-six cases surviving more than five years. *Ann Surg*. 1960;151:571–6. 13805389 10.1097/00000658-196004000-00018 PMC1613585

[5] Carter MF, Dalton DP, Garnett JE. Simultaneous diversion of the urinary and fecal streams utilizing a single abdominal stoma: the double-barreled wet colostomy. *J Urol*. 1989;141:1189–91. 2709507 10.1016/s0022-5347(17)41210-9

[6] Aaronson NK, Ahmedzai S, Bergman B, Bullinger M, Cull A, Duez NJ, et al. The European organization for research and treatment of cancer QLQ-C30: a quality-of-life instrument for use in international clinical trials in oncology. *J Natl Cancer Inst*. 1993;85:365–76. 8433390 10.1093/jnci/85.5.365

[7] Brazier JE, Harper R, Jones NM, O’Cathain A, Thomas KJ, Usherwood T, Westlake L. Validating the SF-36 health survey questionnaire: new outcome measure for primary care. *BMJ*. 1992;305:160–4. 1285753 10.1136/bmj.305.6846.160 PMC1883187

[8] EuroQol–a new facility for the measurement of health-related quality of life. *Health Policy.* 1990;16:199–208. 10.1016/0168-8510(90)90421-9 10109801

[9] Juul K, Prieto L. Quality of life with an intestinal stoma. *Semin Colon Rectal Surg*. 2008;19:167–73.

[10] Brehaut JC, O’Connor AM, Wood TJ, Hack TF, Siminoff L, Gordon E, Feldman-Stewart D. Validation of a decision regret scale. *Med Decis Making*. 2003;23:281–92. 12926578 10.1177/0272989X03256005

[11] Vizzielli G, Tortorella L, Conte C, Chiantera V, Gallotta V, Foschi N, et al. Is a vaginectomy enough or is a pelvic exenteration always required for surgical treatment of recurrent cervical cancer? A propensity-matched study. *Ann Surg Oncol*. 2021;28:3281–90. 33063258 10.1245/s10434-020-09207-w

[12] Limmer AM, Lendzion RJ, Leung C, Wong E, Gilmore AJ. A single-centre experience on the formation of double-barrelled uro-colostomy in pelvic exenteration surgery: a cohort study. *ANZ J Surg*. 2024;94:1161–6. 38193615 10.1111/ans.18856

[13] Nguyen TM, Traeger L, Vather R, Overall B, Cho J, Sammour T. Double-barrelled uro-colostomy versus ileal conduit for urinary diversion following pelvic exenteration: a single-centre experience. *ANZ J Surg*. 2023;93:2450–6. 37132091 10.1111/ans.18498

[14] Wright JP, Guerrero WM, Lucking JR, Bustamante-Lopez L, Monson JRT. The double-barrel wet colostomy: an alternative for urinary diversion after pelvic exenteration. *Surgeon*. 2023;21:375–80. 37087331 10.1016/j.surge.2023.03.004

[15] Lopes de Queiroz F, Barbosa-Silva T, Pyramo Costa LM, Werneck Cortes BJ, Figueiredo JA, Guerra F, et al. Double-barrelled wet colostomy with simultaneous urinary and faecal diversion: results in 9 patients and review of the literature. *Colorectal Dis*. 2006;8:353–9. 16630243 10.1111/j.1463-1318.2006.00952.x

[16] Lago V, Pradillo Aramendi T, Segarra-Vidal B, Padilla-Iserte P, Matute L, Gurrea M, et al. Comparation between the Bricker ileal conduit vs double-barrelled wet colostomy after pelvic exenteration for gynaecological malignancies. *Eur J Obstet Gynecol Reprod Biol*. 2023;282:140–5. 36716537 10.1016/j.ejogrb.2023.01.022

[17] Devulapalli C, Jia Wei AT, DiBiagio JR, Baez ML, Baltodano PA, Seal SM, et al. Primary versus flap closure of perineal defects following oncologic resection: a systematic review and meta-analysis. *Plast Reconstr Surg*. 2016;137:1602–13. 26796372 10.1097/PRS.0000000000002107

[18] Witte DYS, van Ramshorst GH, Lapid O, Bouman MB, Tuynman JB. Flap reconstruction of perineal defects after pelvic exenteration: a systematic description of four choices of surgical reconstruction methods. *Plast Reconstr Surg*. 2021;147:1420–35. 33973948 10.1097/PRS.0000000000007976

[19] McCarthy ASE, Solomon MJ, Koh CE, Firouzbakht A, Jackson SA, Steffens D. Quality of life and functional outcomes following pelvic exenteration and sacrectomy. *Colorectal Dis*. 2020;22:521–8. 31850656 10.1111/codi.14925

[20] Zoucas E, Frederiksen S, Lydrup ML, Mansson W, Gustafson P, Alberius P. Pelvic exenteration for advanced and recurrent malignancy. *World J Surg*. 2010;34:2177–84. 20512493 10.1007/s00268-010-0637-7

[21] Denost Q, Solomon M, Tuech JJ, Ghouti L, Cotte E, Panis Y, et al. International variation in managing locally advanced or recurrent rectal cancer: prospective benchmark analysis. *Br J Surg*. 2020;107:1846–54. 32786027 10.1002/bjs.11854

[22] Austin KK, Young JM, Solomon MJ. Quality of life of survivors after pelvic exenteration for rectal cancer. *Dis Colon Rectum*. 2010;53:1121–6. 20628274 10.1007/DCR.0b013e3181e10c46

[23] Steffens D, Solomon MJ, Young JM, Koh C, Venchiarutti RL, Lee P, Austin K. Cohort study of long-term survival and quality of life following pelvic exenteration. *BJS Open*. 2018;2:328–35. 30263984 10.1002/bjs5.75 PMC6156168

[24] Coker DJ, Koh CE, Steffens D, Young JM, Vuong K, Alchin L, Solomon MJ. The affect of personality traits and decision-making style on postoperative quality of life and distress in patients undergoing pelvic exenteration. *Colorectal Dis*. 2020;22:1139–46. 32180326 10.1111/codi.15036

[25] Young JM, Badgery-Parker T, Masya LM, King M, Koh C, Lynch AC, et al. Quality of life and other patient-reported outcomes following exenteration for pelvic malignancy. *Br J Surg*. 2014;101:277–87. 24420909 10.1002/bjs.9392

[26] Armbruster SD, Sun CC, Westin SN, Bodurka DC, Ramondetta L, Meyer LA, Soliman PT. Prospective assessment of patient-reported outcomes in gynecologic cancer patients before and after pelvic exenteration. *Gynecol Oncol*. 2018;149:484–90. 29622276 10.1016/j.ygyno.2018.03.054 PMC5986607

[27] Guimaraes GC, Ferreira FO, Rossi BM, Aguiar S Jr, Zequi SC, Bachega W, et al. Double-barreled wet colostomy is a safe option for simultaneous urinary and fecal diversion: analysis of 56 procedures from a single institution. *J Surg Oncol*. 2006;93:206–11. 16482600 10.1002/jso.20442

[28] Golda T, Biondo S, Kreisler E, Frago R, Fraccalvieri D, Millan M. Follow-up of double-barreled wet colostomy after pelvic exenteration at a single institution. *Dis Colon Rectum*. 2010;53:822–9. 20389218 10.1007/DCR.0b013e3181cf6cb2

[29] Malone MJ, Izes JK, Hurley LJ. Carcinogenesis: the fate of intestinal segments used in urinary reconstruction. *Urol Clin North Am*. 1997;24:723–8. 9391525 10.1016/s0094-0143(05)70414-6

[30] Spence HM, Hoffman WW, Fosmire GP. Tumour of the colon as a late complication of ureterosigmoidostomy for exstrophy of the bladder. *Br J Urol*. 1979;51:466–70. 534827 10.1111/j.1464-410x.1979.tb03580.x

[31] PelvEx C. Surgical and survival outcomes following pelvic exenteration for locally advanced primary rectal cancer: results from an international collaboration. *Ann Surg*. 2019;269:315–21. 28938268 10.1097/SLA.0000000000002528

