# Supplementary material for: Double-Barrel Urocolostomy After Pelvic Exenteration: Short-Term Morbidity and Patient-Reported Quality of Life
Source: Ann Surg Oncol. 2025 Mar 14;32(6):4534–41. doi: 10.1245/s10434-025-17020-6 (PMC12049299; doi:10.1245/s10434-025-17020-6)
Supplement: Supplementary file 1 — Supplementary file1 (DOCX 26 KB) [file 10434_2025_17020_MOESM1_ESM.docx]

**Supplementary Tables**

*Supplementary Table 1: EORTC QLQ-C30*

| **Scaling (n = 14)** | **Item No.** | **Mean ± SD** | **Range** |
| --- | --- | --- | --- |
| **Global health status / QoL** (higher score indicates better status) | 29, 30 | 77.4 ± 16.2 | 50-100 |
| **Functional scales** (higher score indicates better function) | 1-7, 20-27 | 81.7 ± 10.6 | 66.7-100 |
| Physical functioning | 1 to 5 | 84.3 ± 12.2 | 60-100 |
| Role functioning | 6, 7 | 66.7 ± 25.1 | 16.7-100 |
| Emotional functioning | 21 to 24 | 80.4 ± 17.8 | 41.7-100 |
| Cognitive functioning | 20, 25 | 91.7 ± 10.8 | 66.7-100 |
| Social functioning | 26, 27 | 83.3 ± 13.1 | 66.7-100 |
| **Symptom scales / items** (higher score indicates more problems) | 10-19, 28 | 13.2 ± 12.2 | 0-38.5 |
| Fatigue | 10, 12, 18 | 19.8 ± 17.5 | 0-55.6 |
| Nausea and vomiting | 14, 15 | 2.4 ± 6.1 | 0-16.7 |
| Pain | 9, 19 | 25.0 ± 30.5 | 0-83.3 |
| Dyspnea | 8 | 7.1 ± 14.2 | 0-33.3 |
| Insomnia | 11 | 16.7 ± 36.4 | 0-100 |
| Appetite loss | 13 | 7.1 ± 14.2 | 0-33.3 |
| Constipation | 16 | 9.5 ± 27.5 | 0-100 |
| Diarrhea | 17 | 7.1 ± 14.2 | 0-33.3 |
| Financial difficulties | 28 | 9.5 ± 27.5 | 0-100 |

EORTC, European Organisation for Research and Treatment of Cancer; QLQ, quality-of-life questionnaire; SD, standard deviation; QoL, quality of life

*Supplementary Table 2: EORTC CLC-CR29^a^*

| **Scaling** | **n** | **Item No.** | **Mean ± SD** | **Range** |
| --- | --- | --- | --- | --- |
| Urinary frequency (day, night) | 8, 9 | 31, 32 | 55.7 ± 29.0 | 0-100 |
| Blood and mucus in stool | 11, 9 | 38, 39 | 3.0 ± 10.1 | 0-33.5 |
| Body image | 14 | 45-47 | 24.6 ± 20.6 | 0-55.7 |
| Defecation/stoma problems | 14 | 49-54 | 20.6 ± 20.6 | 0-66.7 |
| Stool frequency/bags change | 14 | 52, 53 | 11.9 ± 24.0 | 0-83.5 |
| **Single-item** |  |  |  |  |
| Urinary incontinence | 7 | 33 | 0 | 0 |
| Dysuria | 7 | 34 | 0 | 0 |
| Abdominal pain | 11 | 35 | 18.1 ± 31.1 | 0-100 |
| Buttock pain | 11 | 36 | 39.3 ± 36.0 | 0-100 |
| Bloated feeling | 12 | 37 | 25.0 ± 35.2 | 0-100 |
| Dry mouth | 12 | 40 | 13.8 ± 30.0 | 0-100 |
| Hair loss | 12 | 41 | 5.5 ± 12.8 | 0-33 |
| Trouble with taste | 12 | 42 | 11.0 ± 16.2 | 0-33 |
| Anxiety | 14 | 43 | 38.1 ± 25.9 | 0-67 |
| Weight | 14 | 44 | 16.6 ± 31.4 | 0-100 |
| Flatulence | 14 | 49 | 30.9 ± 30.6 | 0-100 |
| Fecal incontinence | 14 | 50 | 30.9 ± 38.1 | 0-100 |
| Sore skin around stoma | 14 | 51 | 18.9 ± 16.9 | 0-33 |
| Embarrassed by stoma | 14 | 54 | 19.0 ± 31.3 | 0-100 |
| Stoma care problems | 14 | 55 | 9.5 ± 20.4 | 0-67 |
| **Male** |  |  |  |  |
| Loss of sexual interest | 9 | 56 | 44.3 ± 37.3 | 0-100 |
| Impotence | 8 | 57 | 100 ± 0 | 0 |
| **Female** |  |  |  |  |
| Loss of sexual interest | 3 | 58 | 100 ± 0 | 0 |
| Dyspareunia | 0 | 59 | NA | NA |

EORTC, European Organisation for Research and Treatment of Cancer; QLQ, quality-of-life questionnaire; SD, standard deviation

^a^ higher score indicates more problems

*Supplementary Table 3: SF36 (version 2)^a^*

| **Dimension (n = 14)** | **Item No.** | **Mean ± SD** | **Range** |
| --- | --- | --- | --- |
| Physical functioning (PF) | 3-12 | 75.7 ± 16.2 | 55-100 |
| Role limitations due to physical health (RP) | 13-16 | 50.0 ± 40.7 | 0-100 |
| Role limitations due to emotional problems (RL) | 17-19 | 61.3 ± 30.1 | 0-100 |
| Energy/fatigue (VT) | 23, 27, 29, 31 | 61.1 ± 12.3 | 45-85 |
| Emotional well-being (RE) | 24-26, 28, 30 | 66.9 ± 13.9 | 40-88 |
| Social functioning (SF) | 20, 32 | 72.3 ± 27.4 | 25-100 |
| Pain (BP) | 21, 22 | 74.5 ± 20.7 | 45-100 |
| General health (GH) | 1, 33-36 | 60.4 ± 17.5 | 30-90 |

SD, standard deviation; PF, physical functioning; RP, role limitations–physical; BP, bodily pain; GH, general health; VT, vitality; SF, social functioning; RE, role limitations–emotional; MH, mental health

^a^ higher score indicates more favorable health state

*Supplementary Table 4: EQ-5D-5L*

| **Dimension** | **Item** | **n = 14, n (%)** | |  |
| --- | --- | --- | --- | --- |
| **Mobility** | I have no problems with walking about | 11 (78.6%) | |  |
|  | I have slight problems in walking about | 1 (7.1%) | |  |
|  | I have moderate problems in walking about | 2 (14.3%) | |  |
|  | I have severe problems in walking about | 0 | |  |
|  | I am unable to walk about | 0 | |  |
| **Self-care** | I have no problems washing or dressing myself | 13 (92.9%) | |  |
|  | I have slight problems washing or dressing myself | 0 | |  |
|  | I have moderate problems washing or dressing myself | 1 (7.1%) | |  |
|  | I have severe problems in washing or dressing myself | 0 | |  |
|  | I am unable to wash or dress myself | 0 | |  |
| **Usual activities** | I have no problems doing my usual activities | 6 (42.9%) | |  |
|  | I have slight problems doing my usual activities | 3 (21.4%) | |  |
|  | I have moderate problems doing my usual activities | 4 (28.6%) | |  |
|  | I have severe problems doing my usual activities | 1 (7.1%) | |  |
|  | I am unable to do my usual activities | 0 | |  |
| **Pain / discomfort** | I have no pain or discomfort | 4 (28.6%) | |  |
|  | I have slight pain or discomfort | 7 (50%) | |  |
|  | I have moderate pain or discomfort | 2 (14.3%) | |  |
|  | I have severe pain or discomfort | 0 | |  |
|  | I have extreme pain or discomfort | 1 (7.1%) | |  |
| **Anxiety / depression** | I am not anxious or depressed | 5 (35.7%) | |  |
|  | I am slightly anxious or depressed | 3 (21.4%) | |  |
|  | I am moderately anxious or depressed | 6 (42.9%) | |  |
|  | I am severely anxious or depressed | 0 | |  |
|  | I am extremely anxious or depressed | 0 | |  |
| **VAS score (n = 12), n (SD)** | Score 0-100 (higher score indicates better health) | 72 (15) | |  |
| EQ-5D-5L, EuroQol - five dimensions - five levels; SD, standard deviation; VAS, visual analogue scale | | |  | |
